# A63 THE COLONIC PATHOGEN, *ENTAMOEBA HISTOLYTICA* ACTIVATES CASPASE-4 IN HUMAN MACROPHAGES THAT CLEAVES GASDERMIN D TO FACILITATE IL-1β SECRETION IN THE ABSENCE OF CELL DEATH

**DOI:** 10.1093/jcag/gwab049.062

**Published:** 2022-02-21

**Authors:** S Wang, F Moreau, K Chadee

**Affiliations:** Microbiology and Infectious Diseases, University of Calgary, Calgary, AB, Canada

## Abstract

**Background:**

A hallmark of *Entamoeba histolytica* ( *Eh*) invasion in the gut is acute intestinal inflammation dominated by the secretion of pro-inflammatory cytokines. Live *Eh* in contact with macrophages activates caspase-1 by the recruitment of the NLRP3 inflammasome in a Gal-lectin and *Eh* cysteine proteases 5 ( *Eh*CP-A5)-dependent manner, resulting in the maturation and secretion of IL-1β. *Eh* in contact with macrophages also activates caspase-4 by outside-in signaling but it is unclear how *Eh*-induced caspase-1/4 regulates gasdermin D (GSDMD) cleavage to drive both pore formation and IL-1β secretion without causing cell death. In this study, we interrogated the requirements and mechanism of *Eh*-induced caspase-4 activation in cleaving GSDMD to mediate bioactive IL-1β release.

**Aims:**

Hypothesis: *Eh*-induced activation of caspase-4 regulates GSDMD mediated pro-inflammatory responses.

Specific aim: To quantify caspase-1/4 cleavage of GSDMD in *Eh*-induced pro-inflammatory responses.

**Methods:**

Human PMA-differentiated THP-1 macrophages were used for *Eh*-macrophage studies. Caspase-1/4 activation and GSDMD cleavage were detected by immunoblot analysis. Bioactive IL-1β secretion was quantified by HEK-Blue^TM^ IL-1β reporter cells via the measurement of secreted embryonic alkaline phosphatase (SEAP) and cell pyroptosis (inflammatory cell death) was determined by LDH assay. Immunoprecipitation was performed in HEK 293T cells transfected with human GSDMD plasmid followed by *in vitro* caspase cleavage assay.

**Results:**

Unlike caspase-1, *Eh*-induced caspase-4 activation and IL-1β secretion was independent of the NLRP3 inflammasome as revealed with the use of CRISPR-Cas9 gene edited caspase-1, 4, ASC and NLRP3 macrophages. In the absence of caspase-1, caspase-4 activation was significantly upregulated that promoted the cleavage of GSDMD to induce robust IL-1β secretion. *Eh*-induced caspase-4 played a major role in triggering IL-1β release and GSDMD pore formation as quantified by SEAP assay and immunoprecipitation of overexpressed GSDMD in HEK 293T cells followed by *in vitro* caspase cleavage assay. Pharmacological inhibition of GSDMD pore formation and in CRISPR-Cas9 gene edited GSDMD macrophages, *Eh*-induced IL-1β secretion was highly dependent on GSDMD pore formation and independent of pyroptosis. This was in marked contrast to the positive control, LPS + Nigericin that induced high expression of caspase-1 but not caspase-4 that enhanced GSDMD cleavage and IL-1β secretion and induced massive pyroptosis.

**Conclusions:**

These results suggest that *Eh* induced a state of “hyperactivated macrophages” that led to caspase-4 dependent GSDMD cleavage and IL-1β secretion in the absence of pyroptosis important in disease pathogenesis.

**Funding Agencies:**

NSERC

